# GeNePy3D: a quantitative geometry python toolbox for large scale bioimaging

**DOI:** 10.12688/f1000research.27395.1

**Published:** 2020-11-26

**Authors:** Minh-Son Phan, Anatole Chessel

**Affiliations:** 1 Laboratory of Optics and Biosciences, CNRS, INSERM, Ecole polytechniqe, Institut polytechnique de Paris, Palaiseau, 91120, France

**Keywords:** bioimage informatics, quantitative geometry, computational geometry, workflow, python

## Abstract

The advent of large-scale fluorescence and electronic microscopy techniques along with maturing image analysis is giving life sciences a deluge of geometrical objects in 2D/3D(+t) to deal with. These objects take the form of large scale, localised, precise, single cell, quantitative data such as cells’ positions, shapes, trajectories or lineages, axon traces in whole brains atlases or varied intracellular protein localisations, often in multiple experimental conditions. The data mining of those geometrical objects requires a variety of mathematical and computational tools of diverse accessibility and complexity. Here we present a new Python library for quantitative 3D geometry called GeNePy3D which helps handle and mine information and knowledge from geometric data, providing a unified application programming interface (API) to methods from several domains including computational geometry, scale space methods or spatial statistics. By framing this library as generically as possible, and by linking it to as many state-of-the-art reference algorithms and projects as needed, we help render those often specialist methods accessible to a larger community. We exemplify the usefulness of the  GeNePy3D toolbox by re-analysing a recently published whole-brain zebrafish neuronal atlas, with other applications and examples available online. Along with an open source, documented and exemplified code, we release reusable containers to allow for convenient and wide usability and increased reproducibility.

## Introduction

Bioimage informatics aims at bringing microscopy into quantitative biology, associating higher level information to pixels to answer complex biological questions. In particular machine learning based techniques ^1^ are easing the image analysis step, extracting geometrical objects from multidimensional images. But the next step, transforming that geometrical information into biological knowledge, involves a very diverse set of algorithmic tools in distinct communities, from spatial statistics ^2, 3^ to computational geometry ^4, 5^ or neuroinformatics ^6^. Similarly, the software ecosystem around geometrical data analysis is very diverse and heterogeneous, with reference algorithm implementation spread across languages (Spatstat ^7^ for spatial statistics in R, CGAL ^8^ for computational geometry in C++) or across module in python (scipy ^9^ for generic algorithms, anytree ^10^ for trees, trimesh ^11^ for meshes etc), a lack of generic geometric data exchange format and standard graphical tools like Fiji ^12^ and Icy ^13^ being limited in the flexibility of the analysis easily available. To address this problem, we propose GeNePy3D ^14, 15^, a python library meant as a ’middleware’ library to facilitate building data analysis workflows for geometrical objects by providing one convenient API for geometrical data I/O, conversion and interaction between geometrical objects and access to many common and less common algorithm. We will introduce below the architecture of the library and show one example workflow, re-analysing a published dataset of zebrafish brain neuronal traces by combining traces and brain region to extract quantitative metrics per region.

## Methods

### Architecture

**Figure 1.**
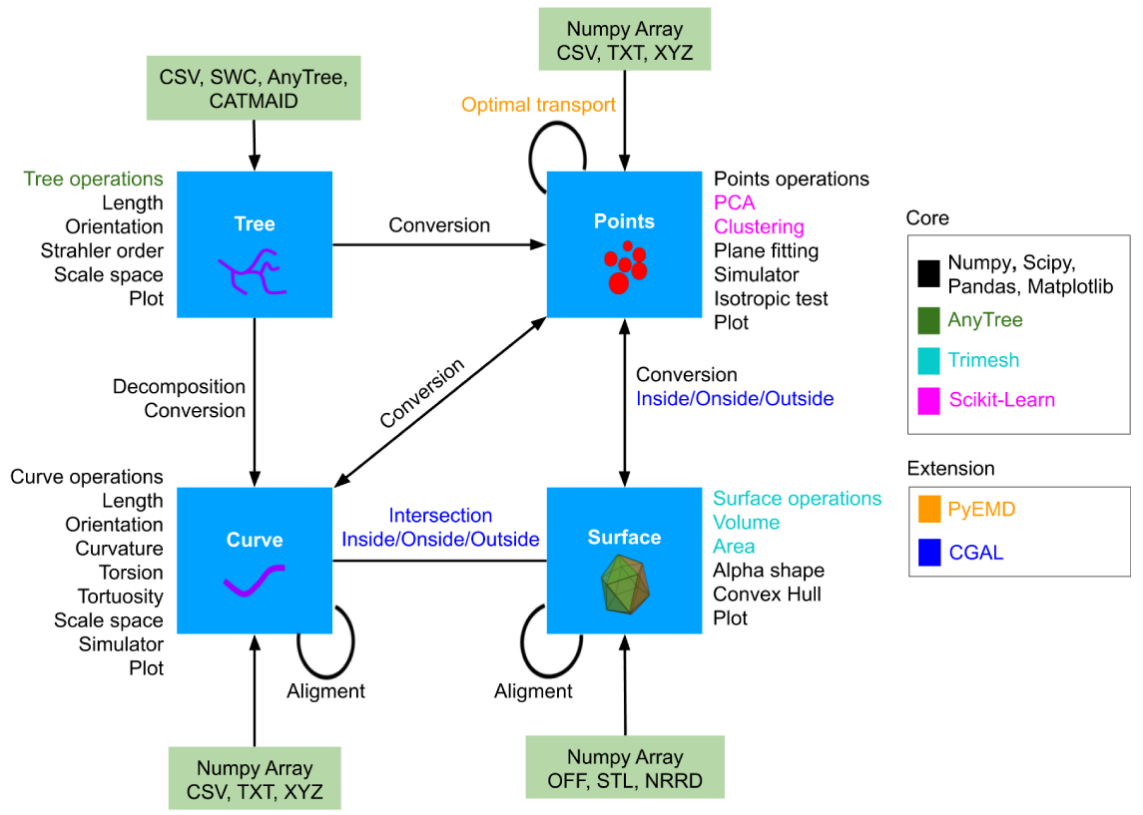
GeNePy3D architecture. The library is structured around four main classes for four principal geometrical objects, and propose various functions acting on them or converting between them, either implemented anew or linking to recognized library.

GeNePy3D ^14, 15^ was designed with any computational-minded life scientist as target user, to provide a simple and homogeneous API. GeNePy3D consists of four main objects ( Figure 1) corresponding to four basic geometrical objects of interest: Points (cells or intracellular object positions...), Curve (particles tracks, neurite branches, microtubules...), Tree (neuronal traces, dividing cell tracks) and Surface (cell surface or other tissue level structure...). Each of them has its own attributes, functions and I/O. We provide ways to transform between them, (decomposing a Tree into sequences of Curve, or converting Points into the Surface that enclose them). Interaction between objects of the same/different classes are also available (alignment of Curves/Surface, optimal transport-based distance between two Points, intersection between Curve and Surface, etc.) Altogether, GeNePy3D offers a unified and seamless way to analyse complex geometrical biological data.

### Implementation

GeNePy3D is implemented in Python, taking advantage of a high-level programming language with simple syntax and many open-source packages. We reused algorithms and functions available from various recognised packages when possible, and developed our own implementation when needed, within a unique interface. Most of the packages we link to are available from the Python package Index (PyPi) and can be easily installed via Python package manager (pip). Figure 1 lists out some functions with colors denoting the package used. Standard ones includes Numpy, Scipy, or Matplotlib, more specific ones includes AnyTree for tree manipulation, TriMesh for surface manipulation or ScikitLearn for machine learning tasks. Other feature are listed as optional, as they come from harder to install or less recognized sources, including the C++ library CGAL, only partially available in Python, for generic object interaction in 3D, or the optimal transport method implemented in PyEMD. Some original development available in GeNePy3d include an algorithm to compute local 3D scale we recently published ^16^. Many common input/output formats are supported including SWC for Tree, CSV, XYZ for Points/Curve and STL and OFF for Surface.

### Operation

GeNePy3D works with Python 3.6. Details of the specific software requirements, documentation including the installation instruction and Python notebooks examples can be accessed via https://genepy3d.gitlab.io. Example pipelines using GeNePy3D are run using Jupyter notebooks. To ease the use and deployment of GeNePy3D we provide ready to use docker containers at https://gitlab.com/genepy3d/genepy3d_dockers.

## Use case

**Figure 2.**
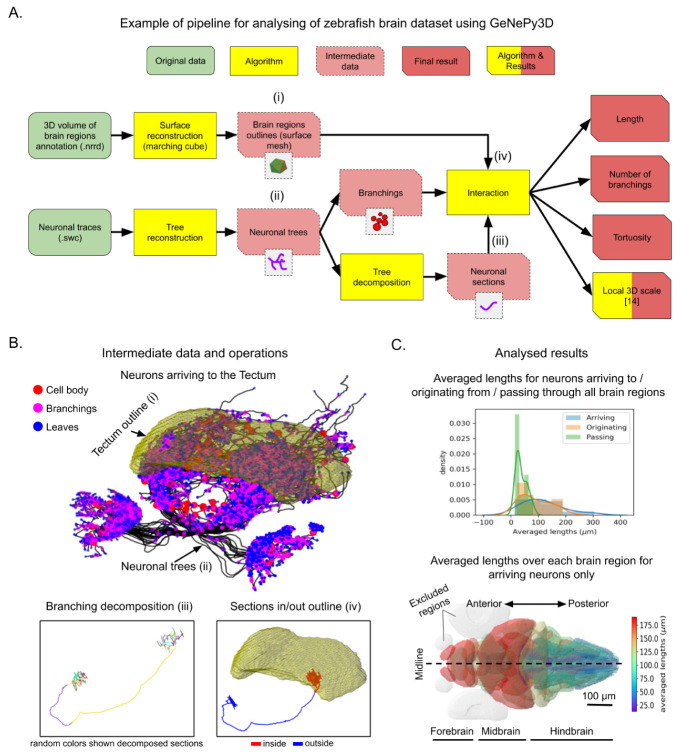
Example workflow for analysing of Larval zebrafish brain dataset ^17^ with GeNePy3D. ( **A**) Workflow schema. ( **B**) Example of intermediate data and operations from the workflow: outline surface of the Tectum and all neurons arriving to it (top), decomposition of a neuronal tree into sections (displayed with random colors) based on branching positions (bottom left), and computing of neuronal sections inside/outside the Tectum (bottom right). ( **C**) Resulting quantifications: distribution of average neuronal lengths for groups of neurons arriving to/originating from/passing all brain regions (top), and heat map of averaged neuronal lengths over each brain region for group of neurons arriving to the brain regions (bottom). The regions with small number of arriving neurons (< 10 neurons) are excluded (in gray). The letters (i-iv) in ( **B**) illustrate some steps in ( **A**).

To exemplify the use of GeNePy3D ^14, 15^, we reanalyzed a recently published dataset containing up to 2000 traced neurons across the whole brain of larval zebrafish ^17^. The authors annotated 36 symmetric regions and established a connectivity atlas for the neurons within these regions. Figure 2A illustrates a possible workflow using GeNePy3D for reanalyzing the dataset. The inputs consist of neuronal traces in SWC formats and a 3D volume in NRRD format containing different annotated labels for the 36 brain regions. The traces are imported into GeNePy3D under Tree objects, while the regions are reconstructed into Surface objects using marching cube algorithm. Figure 2B top illustrates the outline of the Tectum along with all neuronal traces arriving to this brain region. We then extracted branching point positions from the neuronal traces (Tree→Points), decomposed them into sections (Tree→Curves) and checked whether the branching points or curve sections lies within or outside each region (interaction with Surface). Examples of decomposing the traces, computing sections inside and outside the Tectum region are shown in Figure 2B bottom. Finally, we measured within the brain regions neuronal lengths, number of branching points, tortuosities (proportion of length over distance between two end points of the curve), and local 3D scales ^16^ (scale at which the curve transforms to 3D).

Part of the resulting quantification obtained are shown Figure 2C. The top graph shows a longer neuronal length on averaged for groups of neurons arriving to and originating from the regions compared to ones passing through. Figure 2C bottom shows a map of the averaged neuronal length for each brain regions for arriving neurons showing that neurons coming from fore- and midbrain are much longer than those from hindbrain. Detail of all processing steps and additional quantified results can be found at https://gitlab.com/genepy3d/genepy3d_examples/-/tree/master/zebrafish_atlas.

## Conclusions

The advent of machine learning and large scale imaging is leading to large geometrical datasets, and GeNePy3d ^14, 15^ aims at enabling complex analysis workflows based on those objects. But as in other aspects of bioimage informatics, the key will be for the community to work together and define common formats and structures for region of interests and geometric objects to ease the interactions between the various visualisation, data management or analysis tools, and convert raw images to biological knowledge. GeNePy3d is ready to become a component of that ecosystem.

## Data availability

### Source data

The data used for Figure 2 has been published in https://fishatlas.neuro.mpg.de. To download the traces, we choose ’single axons’, ’connect without logging in’, chose ’Kunst *et al.* 2019’ in publications; once all neurons are loaded the download option appears.

## Software availability

**GeNePy3D is hosted at:** https://genepy3d.gitlab.io and easily installable through the PyPi tool.

**Source code available at:** https://gitlab.com/genepy3d.

**Archived source code at time of publication:**

- GeNePy3D: https://doi.org/10.5281/zenodo.4269466 ^14^.
- GeNePy3D_GPL: https://doi.org/10.5281/zenodo.4269484 ^15^.

**License:** The library is distributed as two packages for licensing reasons. The main package GeNePy3D ^14^ is under a BSD 3-Clause Licence, while features that necessitate linking to GPL-licensed code are distributed separately in GeNePy3D_GPL ^15^, under the GNU General Public License v3.0.

The source code for the analysis of Figure 2 is available at https://gitlab.com/genepy3d/genepy3d_examples/-/tree/master/zebrafish_atlas.
