# Supplementary material for: GeNePy3D: a quantitative geometry python toolbox for bioimaging
Source: F1000Res. 2021 Jun 17;9:1374. Originally published 2020 Nov 26. [Version 2] doi: 10.12688/f1000research.27395.2 (PMC8226399; doi:10.12688/f1000research.27395.2)
Supplement: Peer review history

# Peer review: Reviewer response for version 2

**DOI:** 10.5256/f1000research.57818.r87836

**Published:** 2021-06-24

**Authors:** Virginie Uhlmann

**Affiliations:** 1 Wellcome Genome Campus, European Bioinformatics Institute (EMBL-EBI), Cambridge, UK

Thank you for providing in-depth clarifications to all of my points and amending the paper accordingly. I look forward to following up on future GeNePy3D developments.

Are the conclusions about the tool and its performance adequately supported by the findings presented in the article?

Yes

Is the rationale for developing the new software tool clearly explained?

Yes

Is the description of the software tool technically sound?

Yes

Are sufficient details of the code, methods and analysis (if applicable) provided to allow replication of the software development and its use by others?

Partly

Is sufficient information provided to allow interpretation of the expected output datasets and any results generated using the tool?

Yes

Reviewer Expertise:

Bioimage analysis

I confirm that I have read this submission and believe that I have an appropriate level of expertise to confirm that it is of an acceptable scientific standard.

# Peer review: Reviewer response for version 1

**DOI:** 10.5256/f1000research.30274.r80682

**Published:** 2021-03-16

**Authors:** Yizhi Wang

**Affiliations:** 1 Bradley Department of Electrical and Computer Engineering, Virginia Polytechnic Institute and State University, Arlington, Virginia, USA

The author design a Python package to analyze the various geometric objects extracted from microscopy images of the brain. The package combines tools from computational geometry, spatial statistic, and other fields into a unified API. The usefulness of the package is demonstrated by an example of re-analyzing the zebrafish brain region and neuron traces. 

 The tool should be very useful in gaining insights from the microscopy imaging data. The package is adequately documented, and the Docker file makes it easier to use for many users.

Are the conclusions about the tool and its performance adequately supported by the findings presented in the article?

Yes

Is the rationale for developing the new software tool clearly explained?

Yes

Is the description of the software tool technically sound?

Yes

Are sufficient details of the code, methods and analysis (if applicable) provided to allow replication of the software development and its use by others?

Yes

Is sufficient information provided to allow interpretation of the expected output datasets and any results generated using the tool?

Yes

Reviewer Expertise:

Bioimage informatics, machine learning

I confirm that I have read this submission and believe that I have an appropriate level of expertise to confirm that it is of an acceptable scientific standard.

# Peer review: Reviewer response for version 1

**DOI:** 10.5256/f1000research.30274.r75563

**Published:** 2020-12-15

**Authors:** Virginie Uhlmann

**Affiliations:** 1 Wellcome Genome Campus, European Bioinformatics Institute (EMBL-EBI), Cambridge, UK

The authors describe GeNePy3D, a Python toolbox that facilitates the processing and quantification of geometrical objects extracted from images. The goal is for this package to bring together the methods provided in various existing geometrical processing libraries such as PyEMD, Trimesh, or AnyTree. The provided example reanalyzing the larval zebrafish connectome dataset of Kunst *et al. *is well-chosen and convincing. Overall, GeNePy3D is an absolutely relevant software that is likely to be impactful in the bioimage analysis community.

**Major comments:**

- The authors mention Fiji and Icy, which are indeed widely used to quantify geometry from bioimages. They however do not spell out clearly how they envision GeNePy3D to interact with these GUI-based (and Java-based!) alternatives. More details on this aspect should be provided.
- The GitHub repo should be better documented, in particular when it comes to describing the methods used in functions such as a curve or surface alignment (hence the "sufficient details of the code" point above flagged as "partly").
- The article's title emphasizes the "large scale" aspect of GeNePy3D. From the implementation's description, I am under the impression that the scaling ability of this package comes "for free" from the fact that numpy, scipy, pandas, etc all scale extremely well. If there is more and specific efforts have been put into developing methods in a specific manner so as to allow processing of large datasets, this aspect should be discussed in more detail in the implementation section. If not and if this really is simply a consequence of using well-developed Python libraries, I would suggest downplaying a bit the "large scale" aspect of the toolbox. 

**Minor comments:**

- The package name, GeNePy3D, is poorly chosen for two reasons: 1) the meaning of the acronym is unclear, 2) there exists already at least 3 different Python packages called genepy, all containing entirely unrelated algorithms. I would strongly suggest coming up with a more self-descriptive and less overused name.
- I am no expert in software licensing, but I worry that the two-licenses solution adopted here may be unnecessarily confusing for the end-user. Wouldn't there be a way to package the entirety of the library under a single BSD3/GPL license, or license it all under the most restrictive of the two if applicable? If having two repos under two licenses really is the only possible solution, some clear explanation of why this is so should be provided in the article.

Are the conclusions about the tool and its performance adequately supported by the findings presented in the article?

Yes

Is the rationale for developing the new software tool clearly explained?

Yes

Is the description of the software tool technically sound?

Yes

Are sufficient details of the code, methods and analysis (if applicable) provided to allow replication of the software development and its use by others?

Partly

Is sufficient information provided to allow interpretation of the expected output datasets and any results generated using the tool?

Yes

Reviewer Expertise:

Bioimage analysis

I confirm that I have read this submission and believe that I have an appropriate level of expertise to confirm that it is of an acceptable scientific standard, however I have significant reservations, as outlined above.

# Response

**Published:** 2021-06-14

**Authors:** Anatole Chessel

The authors describe GeNePy3D, a Python toolbox that facilitates the processing and quantification of geometrical objects extracted from images. The goal is for this package to bring together the methods provided in various existing geometrical processing libraries such as PyEMD, Trimesh, or AnyTree. The provided example reanalyzing the larval zebrafish connectome dataset of Kunst *et al. *is well-chosen and convincing. Overall, GeNePy3D is an absolutely relevant software that is likely to be impactful in the bioimage analysis community.

*We thank the reviewer for this positive overall assessment.*

**Major comments:**

- The authors mention Fiji and Icy, which are indeed widely used to quantify geometry from bioimages. They however do not spell out clearly how they envision GeNePy3D to interact with these GUI-based (and Java-based!) alternatives. More details on this aspect should be provided.
    

*Indeed Fiji and icy, and more generally the java-based ecosystem of bioimage informatics tools is very important in the community. The python-based ecosystem is becoming very important as well, with developments around napari and scikit image for example. Linking those two ecosystems is very important to allow heterogeneous workflows, and various solutions have been proposed such as https://github.com/imagej/pyimagej ; but we feel this question is generally beyond the scope of this work, which presents a new python library and thus is usable wherever python is usable. For geometrical objects specifically, the development of a common exchange format would go a long way into allowing interactions between genepy3d and the java world, and is definitely part of the roadmap of future development, in genepy3d or elsewhere.*

- The GitHub repo should be better documented, in particular when it comes to describing the methods used in functions such as a curve or surface alignment (hence the "sufficient details of the code" point above flagged as "partly").

*We improved and reformatted the documentation, to make it easier to access and follow. The main documentation, with tutorials and generic information is still at genepy3d.gitlab.io. The reference documentation of the API is now hosted on readthedocs, separately for the two packages: genepy3d.readthedocs.io and genepy3d_gpl.readthedocs.io.*

*Specifically for the alignment functions: that aspect is still essentially lacking in the library as is it, mainly because we had no occasion to implement it. There is one ‘align’ function for curves, which is quite specific and is now better documented. There is a huge bibliography on those topics and many available implementations; it is something that would be very useful to have in the library and that we do plan to tackle in the future. We removed explicit mention of alignment algorithms in the text, to avoid misleading the reader.*

   

- The article's title emphasizes the "large scale" aspect of GeNePy3D. From the implementation's description, I am under the impression that the scaling ability of this package comes "for free" from the fact that numpy, scipy, pandas, etc all scale extremely well. If there is more and specific efforts have been put into developing methods in a specific manner so as to allow processing of large datasets, this aspect should be discussed in more detail in the implementation section. If not and if this really is simply a consequence of using well-developed Python libraries, I would suggest downplaying a bit the "large scale" aspect of the toolbox. 

*The original applications were on large scale images, hence the ‘large scale’ in the title, but it is true that the genepy3d library itself does not provide any specific development for large scale processing. We propose to drop ‘large scale’ from the title to reflect that point, if that is possible at this stage of the publication process.*

**Minor comments:**

- The package name, GeNePy3D, is poorly chosen for two reasons: 1) the meaning of the acronym is unclear, 2) there exists already at least 3 different Python packages called genepy, all containing entirely unrelated algorithms. I would strongly suggest coming up with a more self-descriptive and less overused name.

*Finding a name for a project is complicated and we agree that, in retrospect, better choices most likely exist. It originally stood for Geometry of Neuron in Python in 3D. It might be clearer when heard (something like ‘geeneepaï’). We may indeed change it in the future, if it gains traction and the user and developer base expands; when going out of alpha for example.*

- I am no expert in software licensing, but I worry that the two-licenses solution adopted here may be unnecessarily confusing for the end-user. Wouldn't there be a way to package the entirety of the library under a single BSD3/GPL license, or license it all under the most restrictive of the two if applicable? If having two repos under two licenses really is the only possible solution, some clear explanation of why this is so should be provided in the article.

*We would have welcomed another solution but we do not know of a clean, legal way to mix, in a project under BSD, both GPL and BSD code. Since we want the bulk of the library to be under a BSD license for compatibility with the rest of the python ecosystem (see also this argument for BSD in scientific code: https://www.astrobetter.com/blog/2014/03/10/the-whys-and-hows-of-licensing-scientific-code/), this means corralling out GPL bits. We will try to make them as small as possible, and the added complexity is mitigated by modern python package management, which makes it trivial to install additional packages. We added a sentence to explain this reasoning in the text.*
